# Supplementary material for: Biomolecular condensates form spatially inhomogeneous network fluids
Source: Nat Commun. 2024 Apr 22;15:3413. doi: 10.1038/s41467-024-47602-z (PMC11035652; doi:10.1038/s41467-024-47602-z)
Supplement: Supplementary file 1 — Supplementary Information [file 41467_2024_47602_MOESM1_ESM.pdf]

Supplementary Information for:

Biomolecular condensates form spatially inhomogeneous network fluids

Furqan Dar[1,8], Samuel R. Cohen[1,2,8], Diana M. Mitrea[3], Aaron H. Phillips[4], Gergely Nagy[5], Wellington C. Leite[5], Christopher B. Stanley[6], Jeong-Mo Choi[7,8,*], Richard W. Kriwacki[4,*], Rohit V. Pappu[1,*]

[1]Department of Biomedical Engineering and Center for Biomolecular Condensates, Washington University in St. Louis, St. Louis, MO 63130, USA

[2]Center of Regenerative Medicine, Washington University in St. Louis, St. Louis, MO 63130, USA

[3]Dewpoint Therapeutics Inc., 451 D Street, Boston, MA 02210, USA

[4]Department of Structural Biology, St. Jude Children's Research Hospital, Memphis, TN 38105, USA

[5]Neutron Scattering Division, Oak Ridge National Laboratory, Oak Ridge, TN 37831, USA

[6]Computational Sciences and Engineering Division, Oak Ridge National Laboratory, Oak Ridge, TN 37830

[7]Department of Chemistry and Chemistry Institute for Functional Materials, Pusan National University, Busan 46241, Republic of Korea

[8]These authors contributed equally: Furqan Dar, Samuel R. Cohen, and Jeong-Mo Choi

[*] e-mail: jmchoi@pusan.ac.kr, richard.kriwacki@stjude.org, pappu@wustl.edu

SUPPLEMENTARY FIGURES

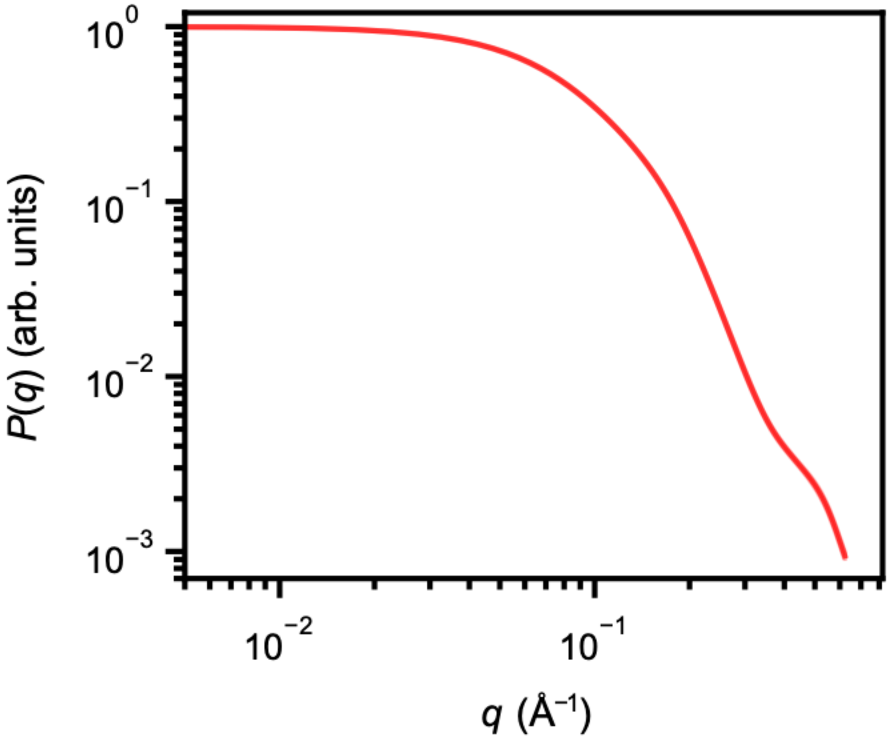

Supplementary Fig. 1: Scattering probability for N130 pentamers, computed using CAMPARI. Snapshots from ABSINTH-based atomistic simulations of N130 in the presence of rpL5 peptides were culled, and the scattering profiles were computed for N130 pentamers, while excluding the rpL5 peptides. The N130 molecules include the oligomerization domains and disordered regions (see Fig. 1a in the main text). Details of how the scattering profiles were computed are as described in the Methods section. Averages were calculated over forty replicates ($n = 40$).

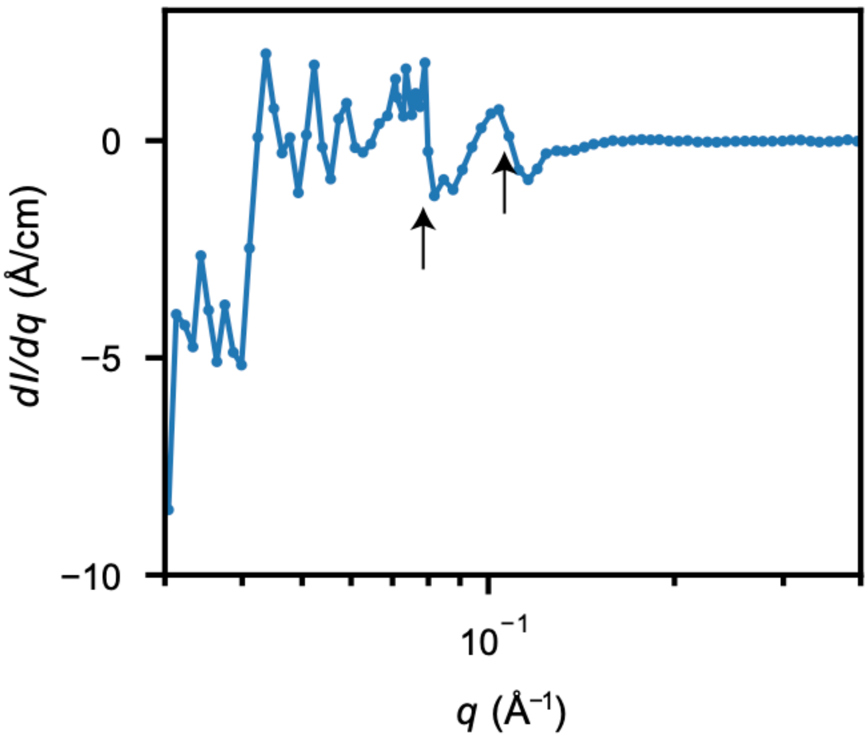

Supplementary Fig. 2: Derivative analysis of the SANS curve for N130+rpL5. The first derivative, dI/dq, of the SANS data confirms the assignment of the two peak positions at large-q corresponding to ~55 Å (right arrow) and ~77 Å (left arrow). Due to low signal-to-noise, unambiguous assignment of additional peaks is not possible using this analysis. The data correspond to a single measurement ($n = 1$).

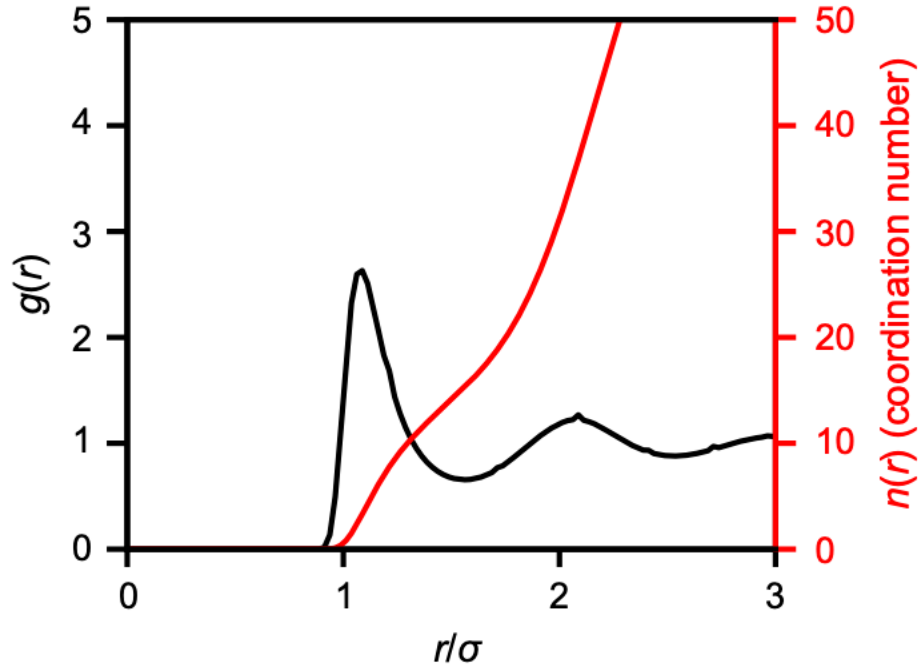

Supplementary Fig. 3: Radial distribution function of a simple liquid. Radial distribution function $g(r)$ showing the correlations between particles in a Lennard-Jones fluid along with the volume integral $n(r)$ quantifying the mean coordination number as a function of interparticle separation r. On average, approximately twelve Lennard-Jones particles are found within the first coordination shell. Averages were calculated over three replicates ($n = 3$).

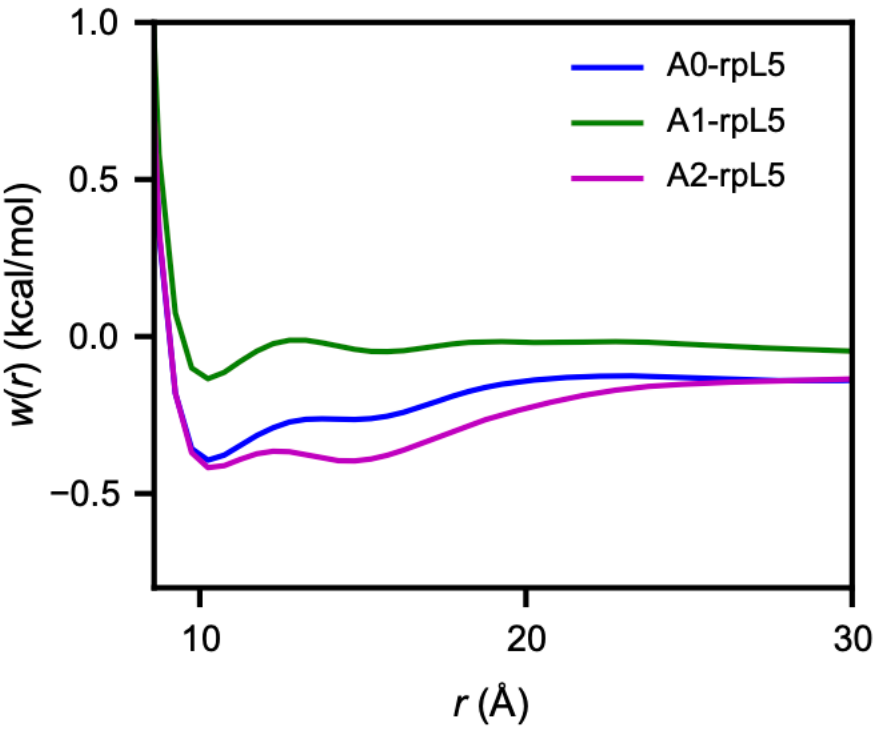

Supplementary Fig. 4: Potentials of mean force calculated with respect to the correlations between the negative charges in the acidic tracts of N130 and the positive charges in rpL5. The potential of mean force was calculated using $w(r) = -RT\ln g(r)$, where R is the gas constant, and T is the simulation temperature. The trend whereby the depths of the first minima follow the pattern A2 < A0 < A1 indicates the interaction hierarchy among the tracts as evident in the corresponding $g(r)$ and the contact map. Averages were calculated over five replicates ($n = 5$).

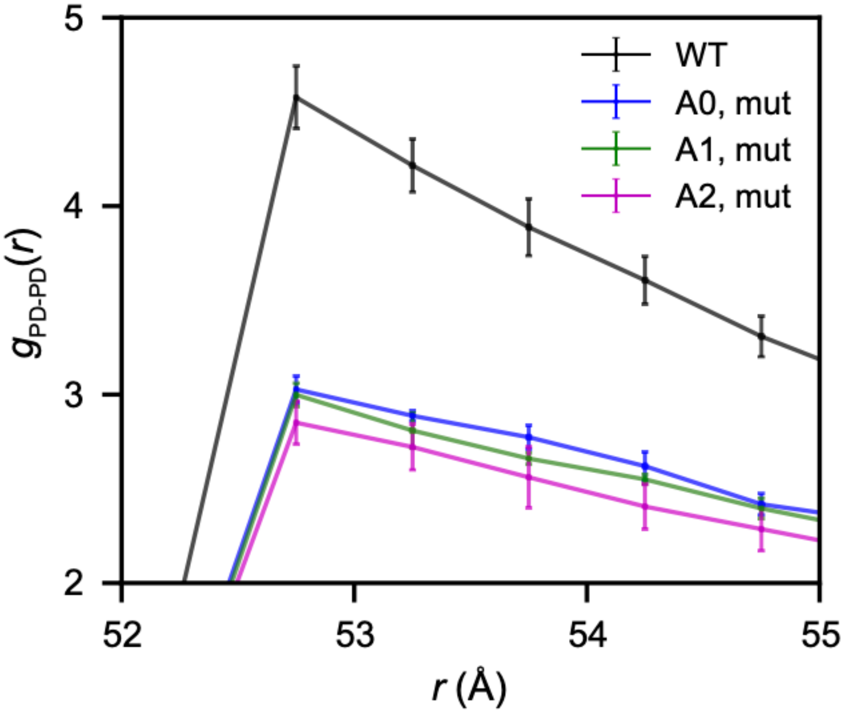

Supplementary Fig. 5: The reduction in the first maximum of $g_{\text{PD-PD}}(r)$ computed after neutralizing the charges in select acidic tracts. The magnitude of the reduction is greatest for the neutralized A2, followed by the A0 and A1 mutants. The values of the peak heights of the A0 and A1 mutants are statistically similar within error. The error bars represent the standard error about the mean over five replicates ($n = 5$).

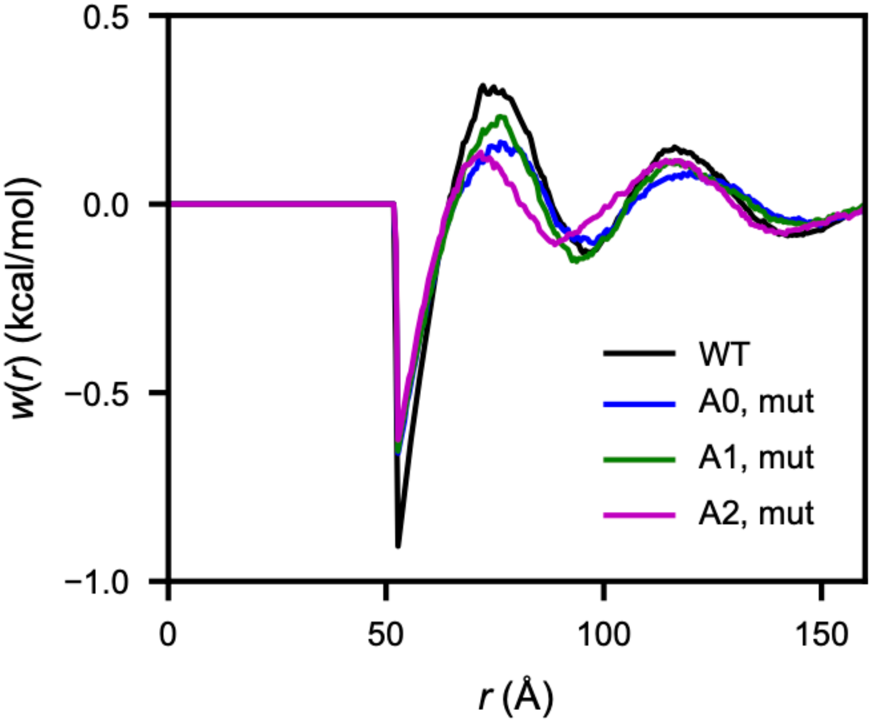

Supplementary Fig. 6: Potentials of mean force calculated with respect to the PD after neutralizing the charges in select acidic tracts. The potential of mean force was calculated as before according to $w(r) = -RT\ln g(r)$, where R is the gas constant, and T is the simulation temperature. The trend at $r{\sim}53$ Å whereby $w(r)$ is less favorable for all acidic tracts compared to wild type, and A2 has the smallest value is also reflected in the corresponding $g(r)$ and the contact map. Averages were calculated over five replicates ($n = 5$).

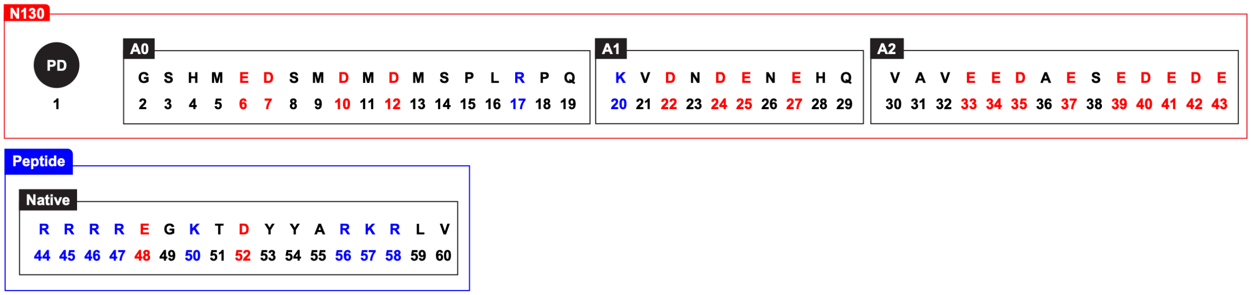

Supplementary Fig. 7: Bead types in the coarse-grained model. The bead types and corresponding amino acid residues are given for the pentamerized domain (PD) and acidic tracts of N130 and the native peptide.

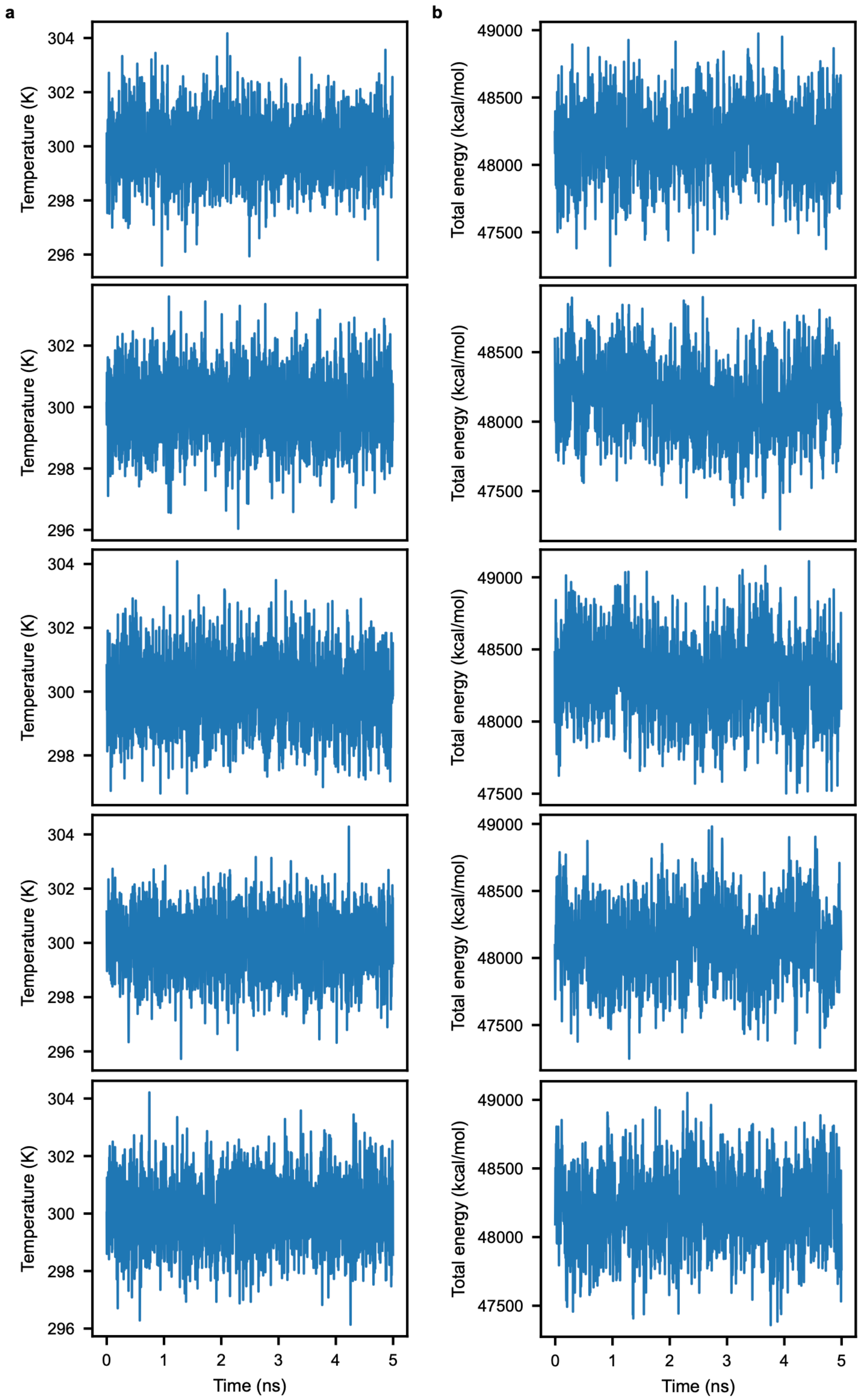

Supplementary Fig. 8: Production runs for the N130 wild type and rpL5 peptides. (a) Temperature vs. time profile for each replicate. **(b)** Energy vs. time profile for each replicate.

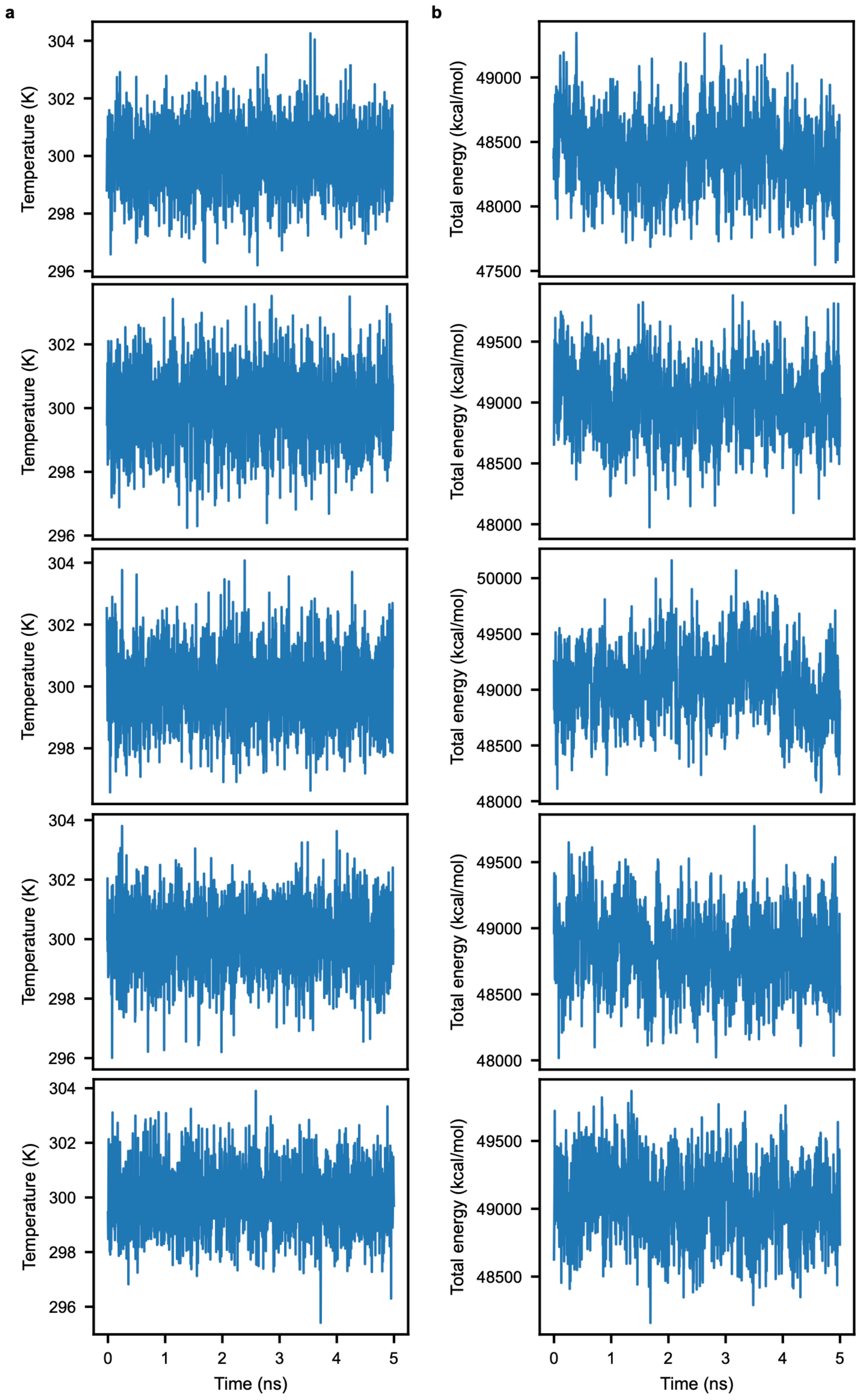

Supplementary Fig. 9: Production runs for the A0 mutants and rpL5 peptides. (a) Temperature vs. time profile for each replicate. **(b)** Energy vs. time profile for each replicate.

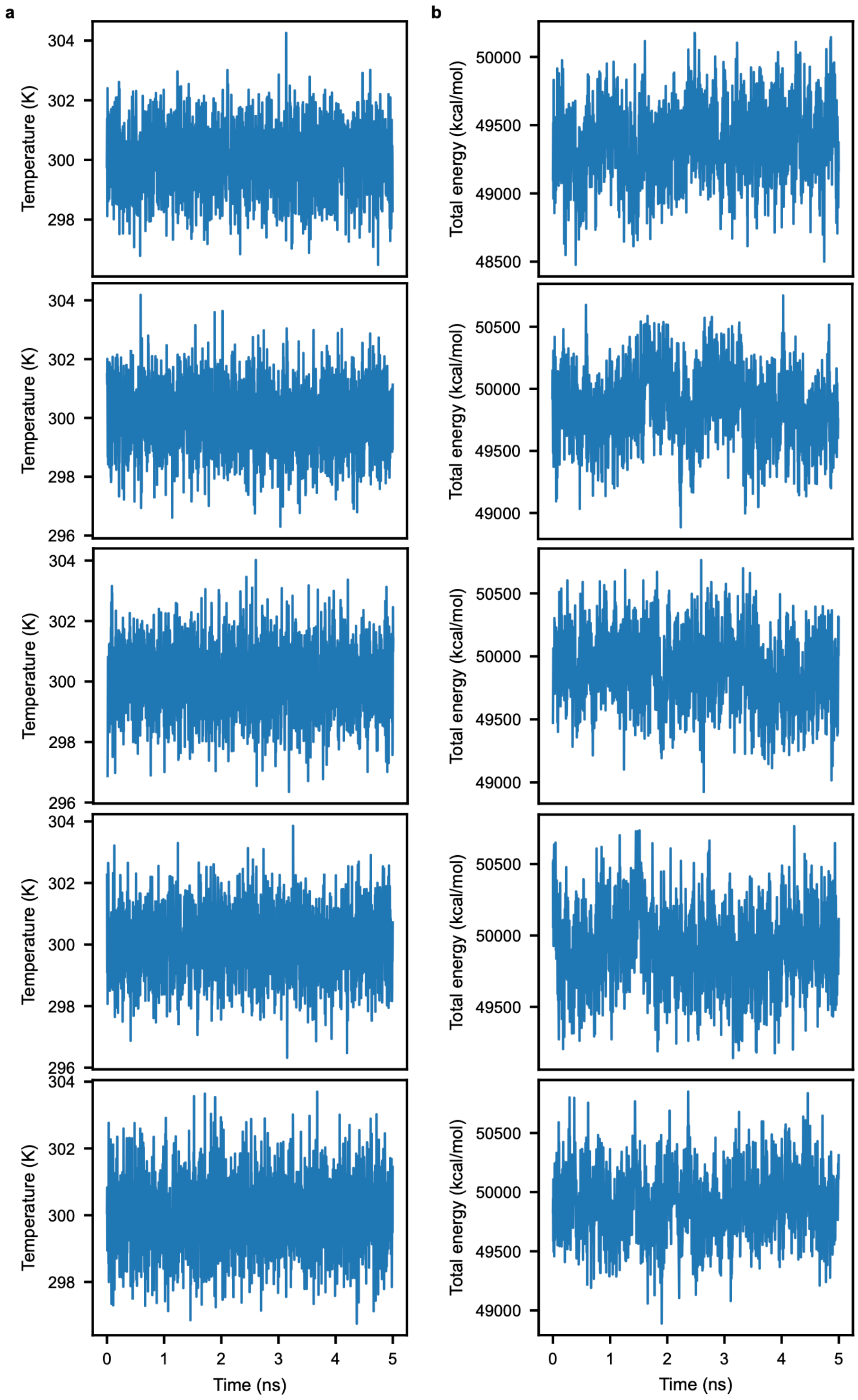

Supplementary Fig. 10: Production runs for the A1 mutants and rpL5 peptides. (a) Temperature vs. time profile for each replicate. **(b)** Energy vs. time profile for each replicate.

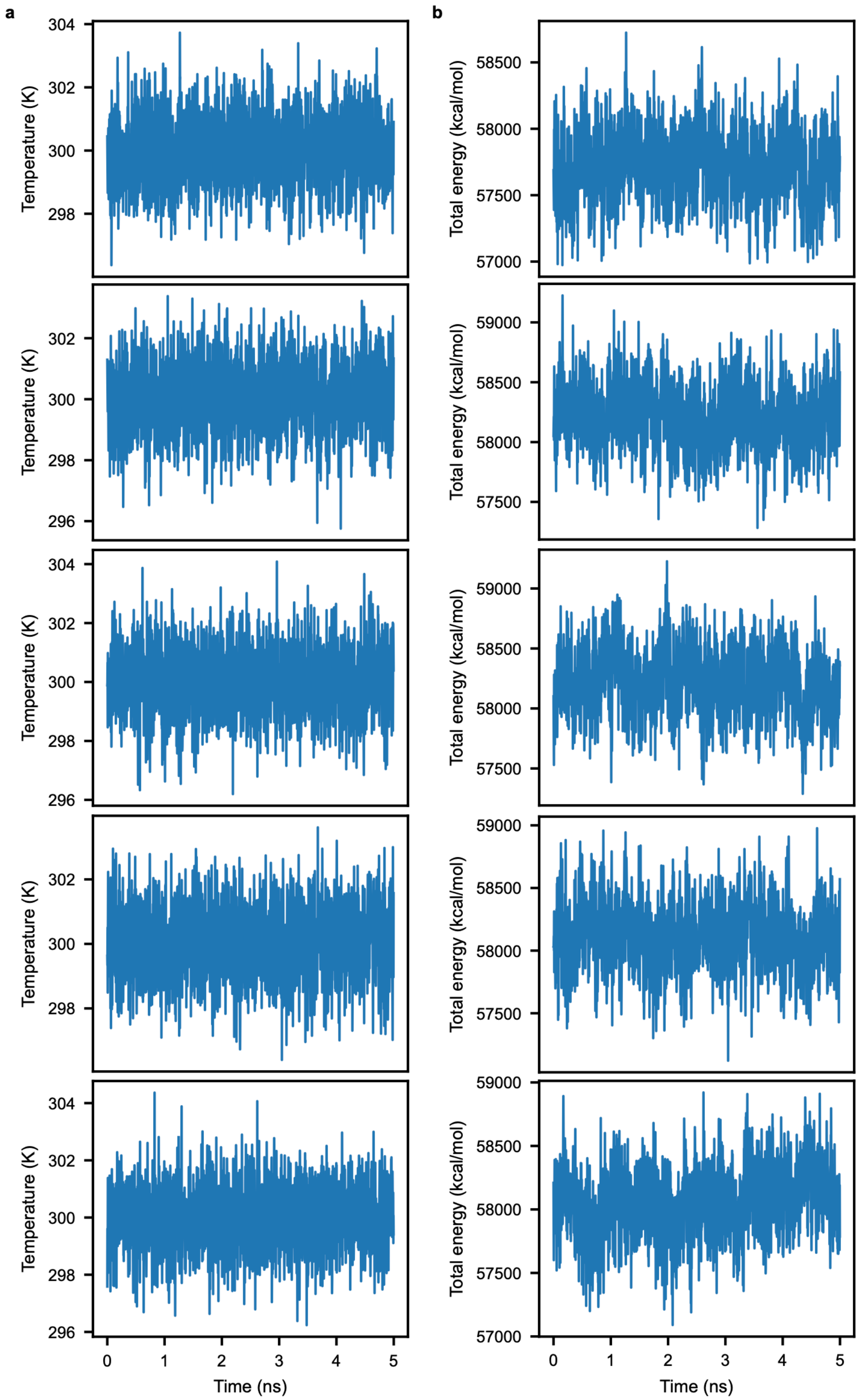

Supplementary Fig. 11: Production runs for the A2 mutants and rpL5 peptides. (a) Temperature vs. time profile for each replicate. **(b)** Energy vs. time profile for each replicate.

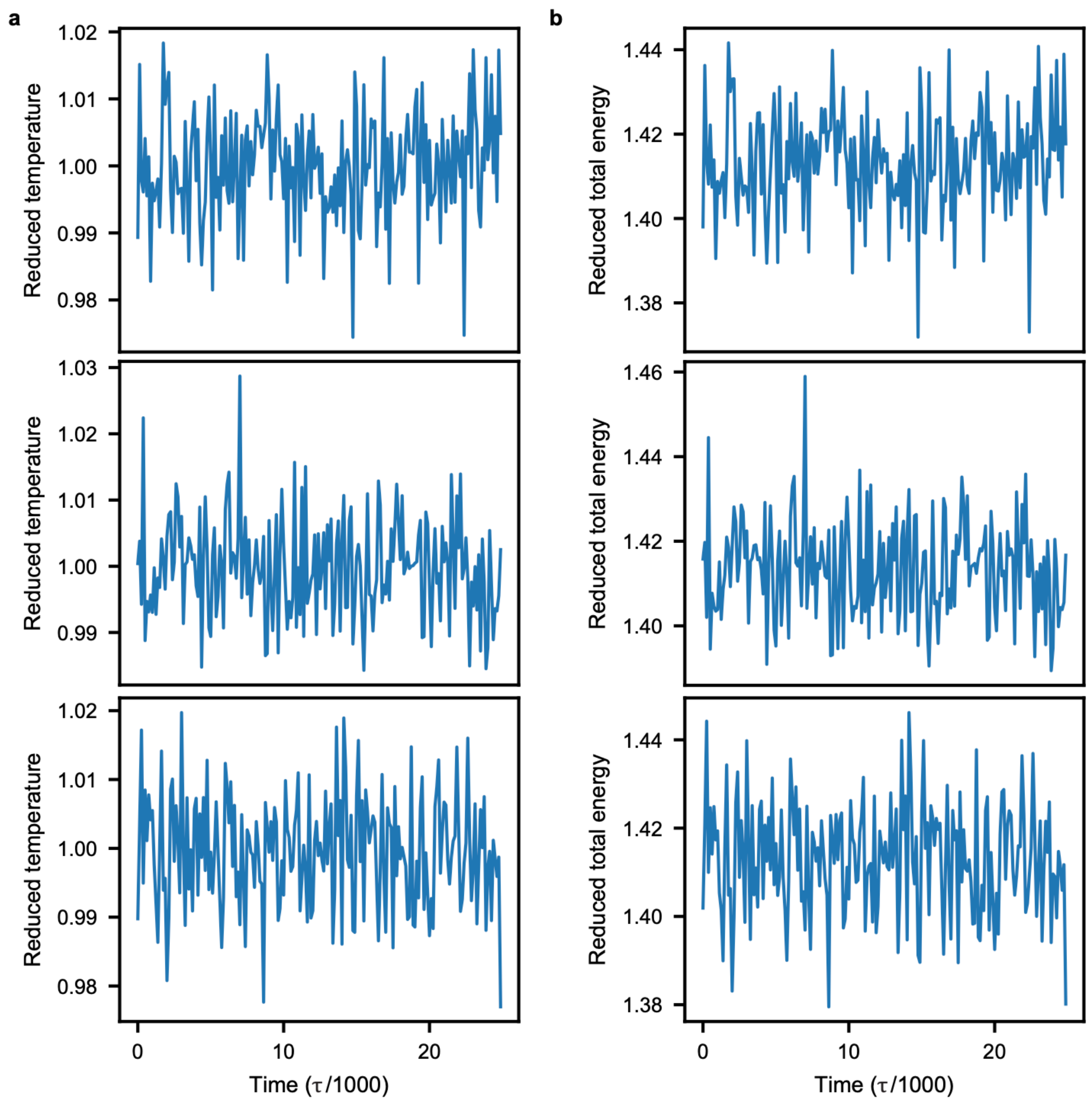

Supplementary Fig. 12: Production runs for the Lennard-Jones gas. (a) Temperature vs. time profile for each replicate. **(b)** Energy vs. time profile for each replicate. The reduced quantities and Lennard-Jones time unit τ are unitless.

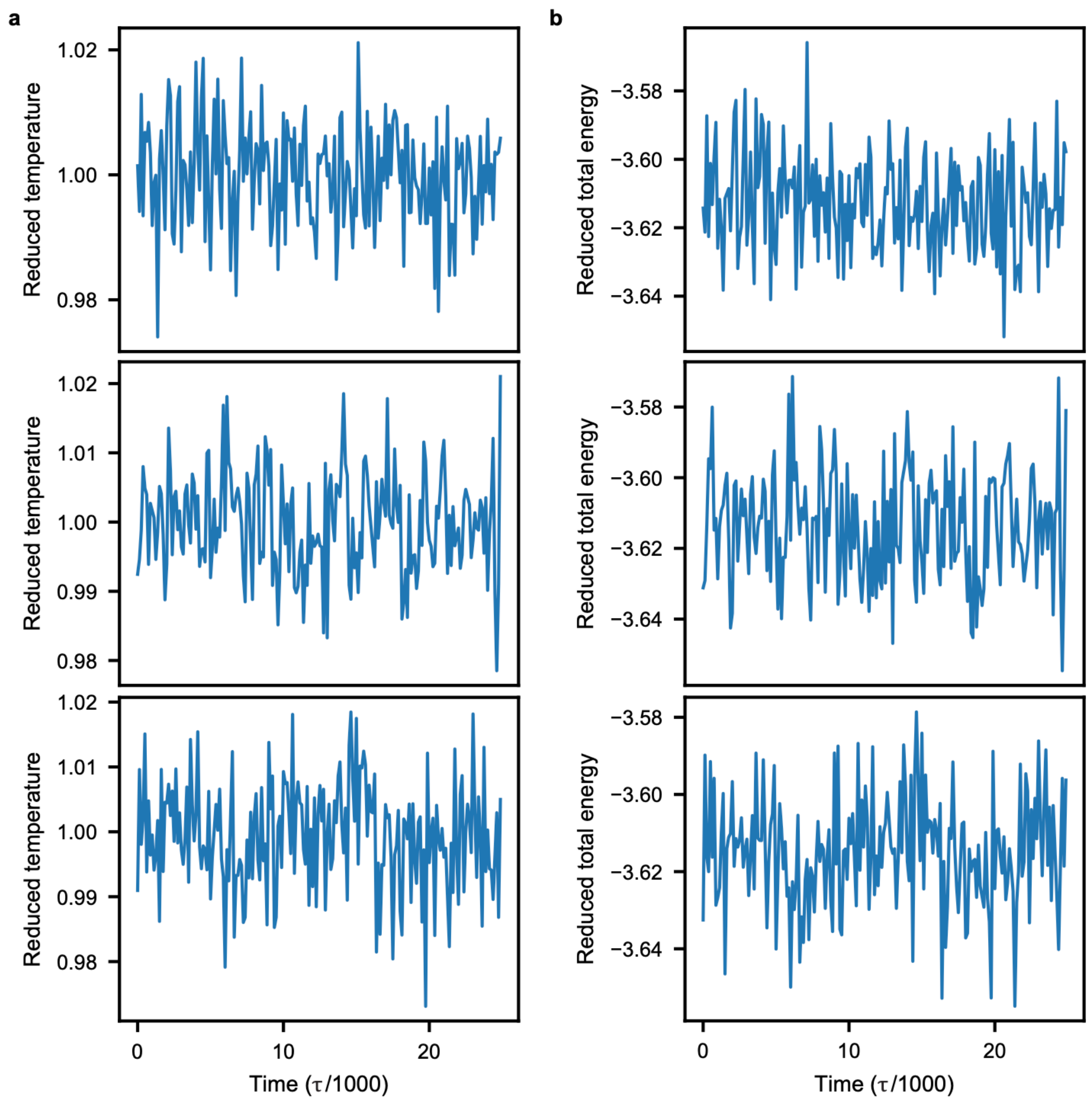

Supplementary Fig. 13: Production runs for the Lennard-Jones fluid. (a) Temperature vs. time profile for each replicate. **(b)** Energy vs. time profile for each replicate. The reduced quantities and Lennard-Jones time unit τ are unitless.

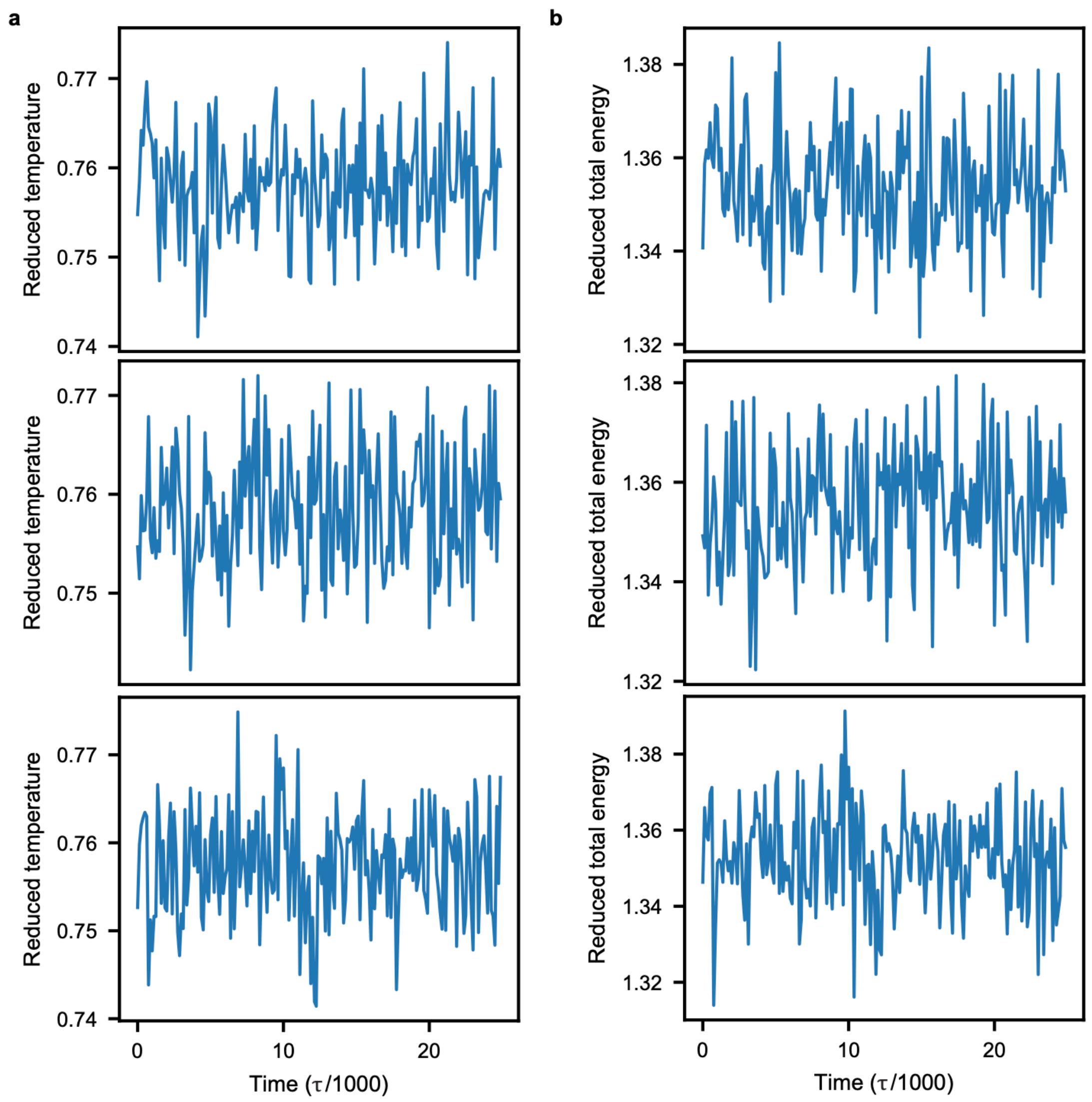

Supplementary Fig. 14: Production runs for the Lennard-Jones solid. (a) Temperature vs. time profile for each replicate. **(b)** Energy vs. time profile for each replicate. The reduced quantities and Lennard-Jones time unit τ are unitless.

SUPPLEMENTARY TABLES

Supplementary Table 1: Details regarding samples, data analysis, modelling, and software used for the SANS measurements. The values for the mean scattering contrast were calculated according to Whitten et al., [1].

(*a*) Sample details			
	**N130**	**N130 + rpl5**	**N130^{+A2} + rpl5**
Organism	**Recombinantly expressed in *E. coli***	**Recombinantly expressed in *E. coli* (N130). Synthetic peptide (rpL5).**	**Recombinantly expressed in *E. coli* (N130^{+A2}). Synthetic peptide (rpL5).**
Source (Catalogue No. or reference)	**N/A**	**N/A**	**N/A**
Description: sequence (including Uniprot ID + uncleaved tags), bound ligands/modifications, *etc.*	**N130 derived from Npm1 (Uniprot P06748).**<br><br>**GSHMEDSMDMDMSP LRPQNYLFGCELKA DKDYHFKVDNDENE HQLSLRTVSLGAGA KDELHIVEAEAMNY EGSPIKVTLATLKM SVQPTVSLGGFEIT PPVVLRLKCGSGPV HISGQHLVAVEEDA ESEDEDE**	**N130^{+A2} derived from Npm1 (Uniprot P06748). rpl5 derived from the full-length protein (Uniprot P46777).**<br><br>**GSHMEDSMDMDMSPL RPQNYLFGCELKADK DYHFKVDNDENEHQL SLRTVSLGAGAKDEL HIVEAEAMNYEGSPI KVTLATLKMSVQPTV SLGGFEITPPVVLRL KCGSGPVHISGQHLV AVEEDAESEDEDE**<br><br>**+**<br><br>**RRRREGKTDYYARKR LV**	**N130^{+A2} derived from Npm1 (Uniprot P06748) and mutations introduced. rpl5 derived from the full-length protein (Uniprot P46777).**<br><br>**GSHMEDEDESEADE ERPQNYLFGCELKA DKDYHFKVDNDENE HQLSLRTVSLGAGA KDELHIVEAEAMNY EGSPIKVTLATLKM SVQPTVSLGGFEIT PPVVLRLKCGSGPV HISGQHLVAVEEDA ESEDEDE**<br><br>**+**<br><br>**RRRREGKTDYYARK RLV**
Extinction coefficient ε (wavelength and units)	**N/A**	**N/A**	**N/A**

Partial specific volume $\bar{\upsilon}$ (cm^3 g^{-1})	**N/A**	**N/A**	**N/A**
Mean solute and solvent scattering length densities and mean scattering contrast $\Delta\bar{\rho}$ (cm^{-2})	**Solute: $3.018 \cdot 10^{10}$ cm^{-2}**<br>**Solvent: $6.377 \cdot 10^{10}$ cm^{-2}**<br>**Contrast:**<br>**$-3.359 \cdot 10^{10}$ cm^{-2}**	**Solute: $3.018 \cdot 10^{10}$ cm^{-2}; $3.819 \cdot 10^{10}$ cm^{-2}**<br>**Solvent:**<br>**$6.377 \cdot 10^{10}$ cm^{-2}**<br>**Contrast: $-3.359 \cdot 10^{10}$ cm^{-2}; $-2.558 \cdot 10^{10}$ cm^{-2}**	**Solute: $3.087 \cdot 10^{10}$ cm^{-2}; $3.819 \cdot 10^{10}$ cm^{-2}**<br>**Solvent: $6.377 \cdot 10^{10}$ cm^{-2}**<br>**Contrast:**<br>**$-3.29 \cdot 10^{10}$ cm^{-2};**<br>**$-2.558 \cdot 10^{10}$ cm^{-2}**
Molecular mass *M* from chemical composition (Da)	**14599.30 Da (monomer)**	**14599.30 Da (monomer); 2221.25 Da**	**14625.02 Da (monomer); 2221.25 Da**
For SEC-SANS, loading volume/concentration, (mg ml^{-1})<br>injection volume (μl), flow rate (ml min^{-1})	**N/A**	**N/A**	**N/A**
Concentration (range/values) measured and method	**200 μM (2.9 mg/ml), Thermo Scientific NanoDrop Spectrophotometer**	**200 μM (2.9 mg/ml); 600 μM (1.3 mg/ml), Thermo Scientific NanoDrop Spectrophotometer**	**200 μM (2.9 mg/ml); 600 μM (1.3 mg/ml), Thermo Scientific NanoDrop Spectrophotometer**
Solvent composition and source	**10 mM Tris, 150 mM NaCl, 2 mM DTT, in D_2O, pH of 7.5**	**10 mM Tris, 150 mM NaCl, 2 mM DTT, in D_2O, pH of 7.5**	**10 mM Tris, 150 mM NaCl, 2 mM DTT, in D_2O pH of 7.5**

(*b*) SANS data collection parameters

Source, instrument and description or reference: **ORNL, SNS, EQ-SANS, 30Hz operation mode. Heller WT, et al. The suite of small-angle neutron scattering instruments at Oak Ridge National Laboratory. J Appl Crystallogr 51, 242-248 (2018).**

Wavelength (Å): **2.5-6.1 and 9.8-13.4 Å**

Beam geometry (size, sample-to-detector distance): **size: 10mm diameter circular, sample-to-detector distance: 4m**

q-measurement range (Å^{-1}): **$0.006 < q < 0.44$**

Absolute scaling method: **porous silica reference**

Basis for normalization to constant counts: **normalization to accelerator total charge**

Method for monitoring radiation damage, X-ray dose where relevant: **N/A**

Exposure time, number of exposures: **2h-2h15min; 1**

Sample configuration including path length and flow rate where relevant: **tumbler, 1mm pathlength**

Sample temperature (ºC): **ambient**

(*c*) Software employed for SANS data reduction, analysis, and interpretation

SANS data reduction to sample–solvent scattering, and extrapolation, merging, desmearing *etc*. as relevant: **as described in: Heller WT, et al. drtsans: The data reduction toolkit for small-angle neutron scattering at Oak Ridge National Laboratory. Softwarex 19, (2022).**

Calculation of ε from sequence: **N/A**

Calculation of $\Delta\bar{\rho}$ and $\bar{\upsilon}$ values from chemical composition: **N/A**

Basic analyses: Guinier, *P*(*r*), scattering particle volume (*e.g.* Porod volume V_P or volume of correlation V_c): **N/A**

Shape/bead modelling: **N/A**

Atomic structure modelling (homology, rigid body, ensemble): **N/A**

Modelling of missing sequence from PDB files: **N/A**

Molecular graphics: **N/A**

(*d*) Structural parameters

Guinier Analysis

$I(0)$ (cm^{-1})	**N/A**
R_g (Å)	**N/A**
q-range (Å^{-1})	**N/A**
Quality-of-fit parameter (with definition)	**N/A**
M from $I(0)$ (ratio to expected value)	**N/A**

P(*r*) analysis

$I(0)$ (cm^{-1})	**N/A**

R_g (Å)	**N/A**
d_{max} (Å)	**N/A**
q-range (Å^{-1})	**N/A**
Quality-of-fit parameter (with definition)	**N/A**
M from *I*(0) (ratio to expected value)	**N/A**
Volume (*e.g.* V_P and/or V_c)	**N/A**
(*e*) Shape modelling results (a complete panel for each method)	
q-range for fitting	**N/A**
Symmetry/anisotropy assumptions	**N/A**
Ambiguity measure(s) with definitions	**N/A**
χ^2 value/range	**N/A**
P value, any other quality-of-fit parameters	**N/A**
Adjustable parameters in the model fit	**N/A**
Model volume and/or *M* estimate	**N/A**
Model precision/resolution	**N/A**
For multiple phase shape models, R_g values and relative phase volumes	**N/A**
(*f*) Atomistic modelling	
Method	**N/A**
q-range for fitting	**N/A**
Symmetry assumptions	**N/A**
Any measures of model precision	**N/A**
χ^2 value/range	**N/A**

P value, any other quality-of-fit parameters	**N/A**
Adjustable parameters in the model fit	**N/A**
Relevant output parameters (*e.g.* predicted R_g/d_{max} values, weights for multi-state models, *etc.*)	**N/A**
Domain/subunit coordinates and contacts, regions of presumed flexibility as appropriate	**N/A**
(*g*) Data and model deposition IDs	
**N/A. No models were generated. The raw scattering data are included in the Source Data file.**	

Supplementary Table 2: LJ parameters for the native rpL5 peptide.

Type 1	Type 2	ϵ (kcal/mol)	σ (Å)	r_c (Å)
1	*	100.0	26.16	26.16
1	1	100.0	52.32	52.32
2	2	0.25	18.66	46.66
3	3	0.25	3.65	9.11
4	4	0.73	5.02	12.54
5	5	0.73	5.95	14.88
6	6	0.2	10.37	25.92
7	7	0.2	10.37	25.92
8	8	0.25	3.61	9.01
9	9	0.73	5.07	12.67
10	10	0.2	10.37	25.92
11	11	0.73	5.10	12.74
12	12	0.2	10.37	25.92
13	13	0.73	5.05	12.63
14	14	0.25	3.60	8.99
15	15	0.25	3.86	9.64
16	16	0.73	4.60	11.49
17	17	0.2	10.37	25.92
18	18	0.25	3.85	9.62
19	19	0.73	4.69	11.72
20	20	0.2	10.37	25.92
21	21	0.73	4.08	10.21
22	22	0.2	10.37	25.92
23	23	0.73	4.20	10.50
24	24	0.2	10.37	25.92

25	25	0.2	10.37	25.92
26	26	0.73	4.24	10.59
27	27	0.2	10.37	25.92
28	28	0.73	4.99	12.46
29	29	0.73	4.67	11.69
30	30	0.73	4.08	10.19
31	31	0.25	3.39	8.47
32	32	0.73	4.08	10.20
33	33	0.2	10.37	25.92
34	34	0.2	10.37	25.92
35	35	0.2	10.37	25.92
36	36	0.25	3.39	8.47
37	37	0.2	10.37	25.92
38	38	0.25	3.61	9.04
39	39	0.2	10.37	25.92
40	40	0.2	10.37	25.92
41	41	0.2	10.37	25.92
42	42	0.2	10.37	25.92
43	43	0.2	10.37	25.92
44	44	0.2	8.5	21.25
45	45	0.2	8.5	21.25
46	46	0.2	8.5	21.25
47	47	0.2	8.5	21.25
48	48	0.2	8.5	21.25
49	49	0.01	2.96	7.39
50	50	0.2	8.5	21.25
51	51	0.01	3.97	9.92
52	52	0.2	8.5	21.25
53	53	0.5	5.64	14.09
54	54	0.5	5.60	14.01
55	55	0.01	3.40	8.50
56	56	0.2	8.5	21.25
57	57	0.2	8.5	21.25
58	58	0.2	8.5	21.25
59	59	0.5	4.60	11.51
60	60	0.5	4.07	10.18

Supplementary Table 3: Bond parameters for the native rpL5 peptide.

Bond type	K (kcal/mol/Å^2)	r_0 (Å)
1	60000	24.17
2	60000	27.43
3	60000	20.63
4	60000	24.50
5	7.11	3.70
6	0.64	4.63
7	0.35	4.69
8	0.72	6.68
9	2.18	5.28
10	2.03	4.40
11	1.08	4.69
12	0.96	5.24
13	1.47	5.31
14	1.07	5.24
15	1.39	5.44
16	0.87	4.49
17	10.47	3.65
18	2.16	4.78
19	0.71	5.85
20	0.97	5.12
21	1.04	5.03
22	1.28	5.07
23	2.31	4.65
24	1.70	4.87
25	1.28	4.81
26	2.40	5.38
27	1.67	5.44
28	1.51	5.26
29	0.72	5.77
30	0.44	5.03
31	6.16	4.03
32	7.61	3.84
33	1.96	5.02
34	1.61	5.61
35	1.35	5.25
36	2.30	4.31
37	2.26	4.73
38	1.92	4.98
39	1.78	4.84
40	1.41	5.25
41	1.39	5.19
42	1.50	5.17

43	1.79	5.29
44	0.57	7.32
45	0.56	6.88
46	0.46	6.97
47	0.68	5.91
48	1.31	4.67
49	1.74	4.57
50	1.42	5.10
51	2.04	4.53
52	0.56	5.86
53	0.20	5.61
54	0.34	4.89
55	1.02	5.48
56	0.72	6.56
57	0.70	6.51
58	0.75	6.06
59	2.38	4.47

Supplementary Table 4: Angle parameters for the native rpL5 peptide.

Angle type	K (kcal/mol)	θ_0 (degs)
1	6000000	38.08
2	6000000	25.58
3	6000000	11.84
4	6000000	44.53
5	2.90	120.66
6	1.46	88.20
7	3.21	92.11
8	4.47	77.38
9	2.47	101.29
10	2.31	113.32
11	5.70	93.42
12	3.42	67.41
13	7.12	98.43
14	3.62	68.99
15	3.95	95.38
16	2.45	113.15
17	5.48	85.38
18	2.50	99.63
19	5.60	74.87
20	3.33	93.10
21	2.71	114.72
22	5.06	72.89
23	5.74	111.86
24	3.55	82.63

25	7.93	78.78
26	5.32	103.35
27	5.42	64.24
28	3.66	96.05
29	2.09	106.70
30	4.28	111.79
31	4.07	80.47
32	3.44	94.31
33	3.02	95.35
34	2.98	121.51
35	5.02	73.45
36	2.89	114.27
37	4.23	78.12
38	2.67	101.09
39	4.43	87.34
40	3.07	98.51
41	4.48	78.73
42	3.03	81.96
43	4.99	72.83
44	3.57	89.60
45	1.70	130.68
46	4.49	80.25
47	2.50	109.36
48	2.14	85.62
49	2.89	84.48
50	2.78	77.81
51	1.66	112.64
52	5.91	67.05
53	2.85	96.20
54	4.34	73.48
55	2.54	97.07

Supplementary Table 5: Quadratic dihedral parameters for the native rpL5 peptide.

Dihedral type	K (kcal/mol)	ϕ_0 (rads)
1	6000000	0.7604
2	6000000	0.3260
3	6000000	2.1276
51	6000000	2.5908
52	6000000	2.2405
53	6000000	2.1536

Supplementary Table 6: Class2 dihedral parameters for the native rpL5 peptide. The mbt, ebt, at, aat, and bb13 parameters are all set to 0.

Dihedral type	K_1 (kcal/mol)	ϕ_{01} (degs)	K_2 (kcal/mol)	ϕ_{02} (degs)	K_3 (kcal/mol)	ϕ_{03} (degs)
4	0.35	192.09	0.17	-36.38	0.05	209.29
5	0.25	88.11	0.38	94.00	0.00	92.25
6	0.90	202.93	0.04	-39.07	0.03	150.19
7	1.17	-3.08	0.21	171.27	0.00	102.96
8	0.19	51.01	0.37	160.82	0.05	253.04
9	0.84	174.34	0.54	45.49	0.00	83.18
10	0.60	201.82	0.22	108.45	0.09	114.99
11	1.01	144.54	0.16	97.05	0.00	93.28
12	1.21	204.55	0.17	140.09	0.00	82.42
13	0.37	129.81	0.13	107.51	0.00	95.26
14	0.73	216.64	0.32	99.59	0.00	91.89
15	0.90	252.26	0.23	134.04	0.33	191.76
16	0.41	150.27	0.29	94.02	0.00	111.68
17	0.21	199.40	0.49	80.89	0.05	0.95
18	0.78	236.42	0.60	143.37	0.25	155.83
19	0.68	197.31	0.25	76.18	0.08	171.15
20	0.99	111.89	0.06	44.15	0.00	106.72
21	1.18	185.92	0.15	42.63	0.05	73.28
22	0.87	5.23	0.64	141.11	0.12	197.37
23	1.40	152.90	0.32	40.89	0.00	89.92
24	1.31	192.95	0.13	102.51	0.05	124.68
25	0.56	76.81	0.34	86.04	0.18	171.77
26	0.74	133.14	0.17	80.98	0.00	92.05
27	0.68	183.81	0.22	15.86	0.03	63.88
28	0.28	15.57	0.40	152.34	0.01	186.48
29	0.14	-19.80	0.44	147.16	0.02	22.56
30	0.77	181.30	0.22	120.81	0.00	185.70
31	0.99	193.81	0.26	85.19	0.01	114.23
32	0.74	154.43	0.16	75.64	0.00	85.07
33	0.97	188.73	0.15	91.70	0.00	141.62

34	0.18	143.51	0.21	160.09	0.05	12.22
35	0.26	200.66	0.36	137.48	0.09	237.57
36	0.00	99.42	0.39	118.04	0.14	83.38
37	0.00	92.68	0.59	163.61	0.43	117.77
38	0.39	-55.81	0.16	84.16	0.08	102.00
39	0.81	193.10	0.20	162.19	0.00	228.93
40	0.41	184.88	0.04	140.44	0.01	0.97
41	0.55	158.94	0.06	94.56	0.06	231.32
42	0.40	184.10	0.35	66.24	0.00	29.64
43	0.59	204.96	0.33	104.71	0.07	140.42
44	0.19	162.15	0.29	102.99	0.04	139.83
45	0.28	198.62	0.37	90.11	0.20	119.32
46	0.24	190.76	0.19	90.03	0.11	79.27
47	0.00	98.76	0.30	83.13	0.15	110.90
48	0.40	23.30	0.32	134.82	0.05	159.42
49	0.28	-36.25	0.36	115.95	0.03	-2.61
50	0.29	175.94	0.23	110.73	0.00	64.62

Supplementary Table 7: Modified LJ parameters for the A0 N130 mutant. The rest of the parameters are the same as the native peptide given in Supplementary Tables 1-5.

Type 1	Type 2	ϵ (kcal/mol)	σ (Å)	r_c (Å)
6	6	0.25	3.39	8.47
7	7	0.25	3.39	8.47
10	10	0.25	3.39	8.47
12	12	0.25	3.39	8.47
17	17	0.25	3.39	8.47

Supplementary Table 8: Modified LJ parameters for the A1 N130 mutant. The rest of the parameters are the same as the native peptide given in Supplementary Tables 1-5.

Type 1	Type 2	ϵ (kcal/mol)	σ (Å)	r_c (Å)
20	20	0.25	3.39	8.47
22	22	0.25	3.39	8.47
24	24	0.25	3.39	8.47
25	25	0.25	3.39	8.47
27	27	0.25	3.39	8.47

Supplementary Table 9: Modified LJ parameters modified for the A2 N130 variant. The rest of the parameters are the same as the native peptide given in Supplementary Tables 1-5.

Type 1	Type 2	ϵ (kcal/mol)	σ (Å)	r_c (Å)
33	33	0.25	3.39	8.47
34	34	0.25	3.39	8.47
35	35	0.25	3.39	8.47
37	37	0.25	3.39	8.47
39	39	0.25	3.39	8.47
40	40	0.25	3.39	8.47
41	41	0.25	3.39	8.47
42	42	0.25	3.39	8.47
43	43	0.25	3.39	8.47
