# Supplementary material for: Biomolecular condensates form spatially inhomogeneous network fluids
Source: Nat Commun. 2024 Apr 22;15:3413. doi: 10.1038/s41467-024-47602-z (PMC11035652; doi:10.1038/s41467-024-47602-z)
Supplement: Supplementary file 3 — Reporting Summary [file 41467_2024_47602_MOESM3_ESM.pdf]

nature portfolio

Corresponding author(s): Jeong-Mo Choi, Richard W. Kriwacki, Rohit V. Pappu

Last updated by author(s): March 19, 2024

Reporting Summary

Nature Portfolio wishes to improve the reproducibility of the work that we publish. This form provides structure for consistency and transparency in reporting. For further information on Nature Portfolio policies, see our Editorial Policies and the Editorial Policy Checklist.

Statistics

For all statistical analyses, confirm that the following items are present in the figure legend, table legend, main text, or Methods section.

n/a	Confirmed	
☐	☒	The exact sample size (*n*) for each experimental group/condition, given as a discrete number and unit of measurement
☐	☒	A statement on whether measurements were taken from distinct samples or whether the same sample was measured repeatedly
☒	☐	The statistical test(s) used AND whether they are one- or two-sided<br>*Only common tests should be described solely by name; describe more complex techniques in the Methods section.*
☒	☐	A description of all covariates tested
☒	☐	A description of any assumptions or corrections, such as tests of normality and adjustment for multiple comparisons
☐	☒	A full description of the statistical parameters including central tendency (e.g. means) or other basic estimates (e.g. regression coefficient) AND variation (e.g. standard deviation) or associated estimates of uncertainty (e.g. confidence intervals)
☒	☐	For null hypothesis testing, the test statistic (e.g. *F*, *t*, *r*) with confidence intervals, effect sizes, degrees of freedom and *P* value noted<br>*Give P values as exact values whenever suitable.*
☒	☐	For Bayesian analysis, information on the choice of priors and Markov chain Monte Carlo settings
☒	☐	For hierarchical and complex designs, identification of the appropriate level for tests and full reporting of outcomes
☒	☐	Estimates of effect sizes (e.g. Cohen's *d*, Pearson's *r*), indicating how they were calculated

Our web collection on statistics for biologists contains articles on many of the points above.

Software and code

Policy information about availability of computer code

Data collection	Coarse-grained MD simulations were done in LAMMPS (16-Dec-2013). All-atom simulations were performed using version 2.0 of CAMPARI and the abs_opls_3.2.prm parameter set. We used CAMELOT (v0.1.2) for generating the coarse-grained models from the all-atom simulations. Input files for the simulations and coordinate files of the final outputs are available via Zenodo (https://zenodo.org/doi/10.5281/zenodo.10823199).
Data analysis	Python (v3.9), VMD (v1.9.3), and MATLAB (r2021b) were used for data analysis. All Source Data and custom-made code for the analysis can be found on GitHub (https://github.com/Pappulab/n130-liquid-structure/). SANS data reduction followed standard procedures using MantidPlot (https://archive.mantidproject.org/) and drtsans (https://code.ornl.gov/sns-hfir-scse/sans/sans-backend).

For manuscripts utilizing custom algorithms or software that are central to the research but not yet described in published literature, software must be made available to editors and reviewers. We strongly encourage code deposition in a community repository (e.g. GitHub). See the Nature Portfolio guidelines for submitting code & software for further information.

Data

Policy information about availability of data

All manuscripts must include a data availability statement. This statement should provide the following information, where applicable:

- Accession codes, unique identifiers, or web links for publicly available datasets
- A description of any restrictions on data availability
- For clinical datasets or third party data, please ensure that the statement adheres to our policy

All data used for generating figures is available on the GitHub repository (https://github.com/Pappulab/n130-liquid-structure/). Raw simulation files are uploaded on the Zenodo repository (https://zenodo.org/doi/10.5281/zenodo.10823199). PDB 4N8M is available from the Protein Data Bank (https://doi.org/10.2210/pdb4N8M/pdb).

Research involving human participants, their data, or biological material

Policy information about studies with human participants or human data. See also policy information about sex, gender (identity/presentation), and sexual orientation and race, ethnicity and racism.

Reporting on sex and gender	N/A
Reporting on race, ethnicity, or other socially relevant groupings	N/A
Population characteristics	N/A
Recruitment	N/A
Ethics oversight	N/A

Note that full information on the approval of the study protocol must also be provided in the manuscript.

Field-specific reporting

Please select the one below that is the best fit for your research. If you are not sure, read the appropriate sections before making your selection.

☒ Life sciences ☐ Behavioural & social sciences ☐ Ecological, evolutionary & environmental sciences

For a reference copy of the document with all sections, see nature.com/documents/nr-reporting-summary-flat.pdf

Life sciences study design

All studies must disclose on these points even when the disclosure is negative.

Sample size	Microscopy measurements were repeated with n=3, giving similar results. A single experiment (n=1) was used for the SANS measurements. The measurements replicate previous findings. The FRAP experiments were performed with n=12, giving recovery times with errors on the order of less than 10%. Five replicates were analyzed for each set of N130+rpL5 simulations, giving reasonable errors, e.g., barely larger than the line width used for the plots in Fig. 6b.
Data exclusions	No data were excluded except a single point, corresponding to 400 μM [rpL5] and 75 μM [N130] in Fig. 1d, due to issues with the noise floor at the phase boundary.
Replication	The SANS data for the system involving the N130 wild type replicate previous findings in the literature (Mitrea et al., eLife 2016) as cited in the main text. Microscopy measurements were repeated in three independent experiments with similar results. Five replicates were analyzed for each set of N130+rpL5 simulations.
Randomization	Randomization is not relevant to our study, which did not use organisms or participants.
Blinding	Blinding is not relevant to our study since observer bias is not an issue.

Reporting for specific materials, systems and methods

We require information from authors about some types of materials, experimental systems and methods used in many studies. Here, indicate whether each material, system or method listed is relevant to your study. If you are not sure if a list item applies to your research, read the appropriate section before selecting a response.

Materials & experimental systems

n/a	Involved in the study
☒	☐ Antibodies
☒	☐ Eukaryotic cell lines
☒	☐ Palaeontology and archaeology
☒	☐ Animals and other organisms
☒	☐ Clinical data
☒	☐ Dual use research of concern
☒	☐ Plants

Methods

n/a	Involved in the study
☒	☐ ChIP-seq
☒	☐ Flow cytometry
☒	☐ MRI-based neuroimaging

Plants

Seed stocks

Report on the source of all seed stocks or other plant material used. If applicable, state the seed stock centre and catalogue number. If plant specimens were collected from the field, describe the collection location, date and sampling procedures.

Novel plant genotypes

Describe the methods by which all novel plant genotypes were produced. This includes those generated by transgenic approaches, gene editing, chemical/radiation-based mutagenesis and hybridization. For transgenic lines, describe the transformation method, the number of independent lines analyzed and the generation upon which experiments were performed. For gene-edited lines, describe the editor used, the endogenous sequence targeted for editing, the targeting guide RNA sequence (if applicable) and how the editor was applied.

Authentication

Describe any authentication procedures for each seed stock used or novel genotype generated. Describe any experiments used to assess the effect of a mutation and, where applicable, how potential secondary effects (e.g. second site T-DNA insertions, mosiacism, off-target gene editing) were examined.
